# Toxoplasmosis Knowledge and Preventive Behaviours Among Pregnant Women in Jeddah, Saudi Arabia: A Cross-Sectional Study

**DOI:** 10.3390/healthcare13020174

**Published:** 2025-01-16

**Authors:** Wejdan T. Alghafari

**Affiliations:** Department of Clinical Nutrition, Faculty of Applied Medical Sciences, King Abdulaziz University, P.O. Box 80324, Jeddah 21589, Saudi Arabia; walghefari@kau.edu.sa

**Keywords:** toxoplasmosis, knowledge, preventive behaviour, pregnant women, Jeddah, Saudi Arabia

## Abstract

**Background:** Toxoplasmosis is an infection caused by the parasite *Toxoplasma gondii* and is considered asymptomatic in most cases. In pregnant women, however, the disease can be transmitted to the foetus, causing severe congenital consequences. Congenital toxoplasmosis can be avoided by practising simple preventive lifestyle measures during pregnancy. **Method:** This cross-sectional study assessed the toxoplasmosis knowledge and preventive behaviours among 135 pregnant Saudi women in Jeddah, Saudi Arabia. Data were collected by face-to-face interviews using a validated questionnaire covering sociodemographic characteristics, knowledge of toxoplasmosis, and preventive behaviours towards toxoplasmosis between January and April 2023 using convenience snowball sampling. SPSS Statistics was used for data analysis and the qualitative parameters were described as frequencies and percentages. **Result:** Approximately 45.2% of the participants displayed a poor knowledge of toxoplasmosis. Moreover, participants aged 31–40 years exhibited significantly higher knowledge compared to those aged less than 30 years. Most participants practised effective preventive behaviours, as demonstrated by 90.4% and 86.7% of them reporting that they habitually washed their hands after handling raw meat and did not eat rare meat, respectively. **Conclusion:** Overall, the poor knowledge of toxoplasmosis among pregnant women in Jeddah highlights the importance of implementing targeted antenatal health education campaigns and interventions to protect pregnant women and minimise the risk of congenital toxoplasmosis.

## 1. Introduction

Toxoplasmosis is a zoonotic disease caused by the obligate intracellular protozoan parasite *Toxoplasma gondii*. Cats are the main definitive hosts for the parasite, while humans and other warm-blooded animals serve as intermediate hosts [1]. Based on the life cycle of *T. gondii*, infection can be acquired in several ways, including the accidental ingestion of parasite oocysts shed in the faeces of infected cats, which may contaminate water, food, or soil. Another important transmission route is the consumption of raw or undercooked meat containing the parasite bradyzoites. Furthermore, *T. gondii* can be transmitted vertically via tachyzoites that pass through the placenta to the foetus [2]. Infections are rarely transmitted through blood transfusion or organ transplantation [3]. Moreover, recent research reported that the epidemiology of *T. gondii* transmission has changed over time, with outbreaks now more commonly linked to contaminated water, vegetables, and fruits [4].

Toxoplasmosis in pregnant women and otherwise healthy individuals is considered asymptomatic [5]. In some cases, pregnant women may experience mild symptoms such as malaise, lymphadenopathy, and fever [6]. Nevertheless, the consequences of congenital infections can be dangerous for the foetus. Indeed, congenital toxoplasmosis may result in visual or neurological impairments, miscarriage, or even foetal death [7].

Toxoplasmosis prevalence is significantly affected by several factors, including geographic location, climate, environmental conditions, dietary habits, and sociodemographic characteristics [8]. A high prevalence of toxoplasmosis has been reported among pregnant women and women of childbearing age from various countries in the Middle East and South America, as well as other low-income countries [8]. Moreover, studies have reported seroprevalence rates among pregnant women of 49.0% in Malaysia [9], 28.3% in Thailand [9], and 23.8% in the Philippines [10].

A comprehensive systematic review of studies performed in Saudi Arabia reported a high seroprevalence of *T. gondii* infection among Saudi Arabian women of childbearing age [11]. Previous studies have indicated that the prevalence of toxoplasmosis varies greatly among the different governorates of the Kingdom of Saudi Arabia, with Jeddah being recorded as having the highest prevalence at 61.4% [12], followed by Al Ahsa at 51.4% [13], Riyadh at 38.0% [14], and Makkah at 35.6% [15]. However, the incidence of congenital toxoplasmosis in Saudi Arabia remains largely unknown and most available information originates from regional studies. A recent work reported that the seroprevalence of *T. gondii* infection among Saudi babies residing in the Jeddah Region was 18.53% [16]. Another recent study conducted in the Eastern Province of Saudi Arabia reported seropositivity rates of 21.0% and 0.8% for anti-*T. gondii* IgG and IgM antibodies, respectively, in 500 paired maternal/cord blood samples collected during delivery [17].

The variation in toxoplasmosis seroprevalence across different regions of Saudi Arabia can be attributed to several factors, such as climate, contact with animals, and dietary habits. Notably, higher prevalence rates have been reported in rural areas, which can be explained by individuals in these areas often having closer contact with livestock and a higher likelihood of consuming less thoroughly cooked meats [15]. Understanding these factors is crucial for developing targeted prevention strategies and public health interventions tailored to specific regional contexts within Saudi Arabia.

A recent review article highlighted a widespread lack of knowledge about toxoplasmosis among pregnant women globally. The study reported that 66% of Brazilian women and 72% of women worldwide have an insufficient understanding of the disease [18]. Studies conducted in the United States [19], Brazil [20], Morocco [21], and several Asian countries including Malaysia, the Philippines, and Thailand [22] have consistently reported a poor knowledge of toxoplasmosis among pregnant women. Nonetheless, most women in these studies were found to practise suitable preventive behaviours. By contrast, a study performed in Kuwait [23] revealed both inadequate knowledge and insufficient preventive behaviours towards toxoplasmosis among Kuwaiti pregnant women, highlighting regional variations in knowledge and practices.

In Saudi Arabia, the landscape of toxoplasmosis awareness among pregnant women remains underexamined, with only two studies published to date. A study conducted in Al Ahsa [24] and another in Dhahran [25] both reported insufficient knowledge and a lack of necessary preventive behaviours among pregnant women. These findings indicate that there exists a critical gap in toxoplasmosis knowledge and preventive behaviours within the Saudi healthcare context, emphasising the need for further research and targeted interventions in the kingdom.

Toxoplasmosis poses a significant public health challenge, particularly for pregnant women. Despite its known detrimental effects on foetal health and its high prevalence in Jeddah, no previous studies have assessed the knowledge and preventive behaviours among pregnant women in this city. This lack of local data creates a critical gap in our understanding of toxoplasmosis awareness and prevention practices in a high-risk area. Consequently, the cultural, educational, and socioeconomic factors that may influence toxoplasmosis knowledge and preventive behaviours among pregnant women in Jeddah remain poorly understood. This knowledge gap impedes the development of the targeted public health interventions and population-specific educational campaigns that are crucial for effectively addressing this pressing health concern in the region. Therefore, the aim of this study was to evaluate the knowledge and preventive behaviours relating to toxoplasmosis among pregnant women in Jeddah, Saudi Arabia.

## 2. Materials and Methods

### 2.1. Study Design and Ethical Approval

This cross-sectional study was conducted at three obstetrics and gynaecology clinics in Jeddah, Saudi Arabia, including two private clinics and one government clinic. Ethical approval was obtained from the Biomedical Ethics Research Committee of King Abdulaziz University Hospital (reference no. 124-19).

### 2.2. Participants and Recruitment

In total, 135 women were recruited between January and April 2023 using convenience snowball sampling. Women were eligible to participate if they were Saudi, pregnant at any gestational stage, and aged 20 years or older. Women were excluded if they were non-Saudi, non-pregnant, under 20 years of age, or unwilling to participate in the study. The researcher approached pregnant women who were present at the three clinics during the designated data collection periods. This included women waiting for their appointments and those who had just completed their consultations, depending on what was least disruptive to their care. The women were invited to participate in the study, and those who agreed and met the inclusion criteria were enrolled. The researcher then used a validated Arabic questionnaire to conduct face-to-face interviews with the participants. The researcher also provided instructions regarding the proposed study and read the consent form to the participants before they agreed to participate.

### 2.3. Sample Size Calculation

According to the Saudi General Authority for Statistics, the number of pregnant Saudi women in Makkah Province was 67,407 in 2020 [26]. The required sample size (*n*) was determined to be 84 at a statistical power of 80%, an error margin of 7%, and a design effect of 1 using the Epi Info online sample size calculator developed by the Division of Health Informatics and Surveillance and the Center for Surveillance, Epidemiology, and Laboratory Services of the U.S. Centers for Disease Control and Prevention.

### 2.4. Study Questionnaire

The questionnaire was adapted from a previously published validated study [19]. To cater to the target population, the questionnaire was translated from English to Arabic using the Brislin back-translation method to ensure accuracy [27]. The Arabic questionnaire was subsequently validated by two experts to ensure cultural appropriateness and relevance for Saudi women (face and content validity) and the questions were modified accordingly. A pilot test was performed with 15 pregnant Saudi women who were not included in the main sample set to assess comprehension and suitability, and minor adjustments were made based on the obtained feedback. Reliability was evaluated by performing a test/retest study with an average of two weeks between the two surveys, where the average coefficient of correlation was found to be 0.83.

The questionnaire comprised three sections. The first section contained nine questions regarding the sociodemographic characteristics of the participants, including their age, educational level, profession, trimester of pregnancy, number of previous deliveries, history of abortion, whether they had ever seen, heard, or read any information about toxoplasmosis, whether they had ever been tested for toxoplasmosis, and whether they had ever owned cats.

The second section assessed the participants’ knowledge of toxoplasmosis using 24 questions covering four areas: (i) general information on toxoplasmosis (4 questions), (ii) risk factors for toxoplasmosis (3 questions), (iii) symptoms and timing of infection (10 questions), and (iv) preventive knowledge (7 questions).

The third section examined the preventive behaviours towards toxoplasmosis practised by the participants since becoming pregnant and comprised four questions: whether they considered it important to habitually wash their hands after gardening, whether they considered it important to habitually wash their hands after changing cat litter, whether they habitually washed their hands after handling raw meat, and whether they ate rare meat.

A score of ‘1′ was assigned for every right answer, whereas a score of ‘0′ was assigned for every wrong answer and when the participant responded ‘not sure’. Each participant’s percentage and overall score were then calculated. The overall percentage knowledge score was categorised as ‘poor’ (less than 50%), ‘fair’ (50% to less than 70%), or ‘good’ (70% or more). For each preventive behaviours question, participants’ responses were categorised as ‘yes’, ‘no’, or ‘not sure’, with ‘yes’ indicating adherence to the preventive behaviour.

### 2.5. Statistical Analysis

SPSS Statistics (version 28; IBM Corp., Armonk, NY, USA) was used for data entry and statistical analysis. The qualitative parameters were described as frequencies and percentages. Links between toxoplasmosis and categorical variables were evaluated by chi-square tests, and a *p* value of less than 0.05 was considered to indicate statistical significance. Univariate and multivariate analyses were performed using the crude odds ratio (OR) for univariate analysis and the adjusted odds ratio (aOR) for multivariate analysis. To assess statistical significance, 95% confidence intervals (CIs) were utilised for both the crude and adjusted odds ratios. Associations were considered statistically significant when the 95% CI did not include 1.

## 3. Results

### 3.1. Sociodemographic Characteristics

A total of 135 pregnant women participated in this study (response rate: 97.8%). The sociodemographic characteristics of the participants are summarised in Table 1. The majority of participants (57.0%) were aged ≤30 years, and 34.8% were aged between 31 and 40 years. Most participants (74.1%) were educated to university level, and more than half (59.3%) were housewives. More than half of the participants (57.0%) were in their third trimester, and 38.5% reported a history of abortion. Less than half of the participants (46.7%) had ever seen, heard, or read any information about toxoplasmosis. Only 14.8% of the participants had ever been tested for toxoplasmosis, and only 8.1% reported ever having owned cats.

### 3.2. Knowledge of Toxoplasmosis

The knowledge of toxoplasmosis among the participants is summarised in Table 2. Of the 135 participants, almost half (48.9%) correctly answered that infection is the cause of toxoplasmosis, while 50.4% correctly answered that it is not caused by a poison. Approximately three-fifths of the participants (60.7%) thought that *T. gondii* is shed in the faeces of infected cats, and only 32.6% believed that the parasite could be found in raw or undercooked meat.

Regarding the knowledge of toxoplasmosis risk factors, 57.8% of the participants thought that people could become infected with toxoplasmosis by changing cat litter, whereas only 4.4% recognised that infection could be caused by eating undercooked meat and 28.1% knew that it could be caused by gardening without gloves.

With respect to the knowledge of toxoplasmosis symptoms and the timing of infection, most participants (68.9%) were aware that pregnant women could develop serious complications after acquiring toxoplasmosis; however, only 42.2% knew that unborn and/or newborn children could develop serious complications. Approximately half of the participants (54.1%) were aware that toxoplasmosis can cause fever and flu-like symptoms. Only 17.8% of the participants knew that toxoplasmosis may cause swollen glands, and 41.5% knew that toxoplasmosis could be symptomless in pregnant women. Moreover, only 31.1% of participants were aware that toxoplasmosis is rarely passed from a pregnant woman to her foetus if infection occurred prior to her becoming pregnant, whereas 54.8% of participants knew that *T. gondii* infection could only be passed from a pregnant woman to her foetus if she was newly infected during the pregnancy. Additionally, only 32.6% knew that newborns infected with toxoplasmosis could be free of symptoms at birth but may develop them later in life, and only 22.2% knew that such babies could suffer from vision problems. Moreover, approximately two-thirds of participants (67.4%) knew that a baby infected with *T. gondii* could be treated using medication.

In terms of knowledge about the prevention of toxoplasmosis, the majority of participants believed that avoiding stray cats (82.2%), ensuring that a cat’s litterbox is changed daily (74.1%), and letting someone else change a cat’s litterbox (69.6%) were preventive measures against toxoplasmosis. Moreover, the majority believed that cooking meat well until no pink is seen and the juices run clear (60.0%), thoroughly washing and/or peeling all fruits and vegetables before eating them (59.3%), and cleaning all cutting boards and utensils thoroughly after each use (60.0%) can also prevent infection.

Overall, 45.2% of the participants exhibited poor knowledge about toxoplasmosis, 32.6% had fair knowledge, and 22.2% displayed good knowledge.

### 3.3. Associations Between Sociodemographic Factors and Knowledge of Toxoplasmosis

The associations between sociodemographic factors and knowledge of toxoplasmosis are presented in Table 3. Approximately one-third (34.0%) of participants in the 31–40-year age group, compared with only 9.1% of those aged 41–50 years, displayed a good level of knowledge about toxoplasmosis (*p* = 0.049). Women who had experienced 4–7 previous deliveries exhibited a significantly higher level of knowledge about toxoplasmosis than the other groups (*p* = 0.044). Multivariate logistic regression analysis revealed that compared with the women aged ≤30 years, those aged 31–40 years were less likely to express poor/fair knowledge about toxoplasmosis (aOR = 0.38; 95% confidence interval = 0.16–0.89). None of the other factors examined were significantly linked with the level of knowledge about toxoplasmosis.

### 3.4. Toxoplasmosis Preventive Behaviours During Pregnancy

The self-reported preventive behaviours practised by the participants since becoming pregnant are summarised in Table 4. The majority of participants (90.4%) reported that they consider it important to habitually wash their hands after handling raw meat, while 86.7% reported that they did not eat rare meat. By contrast, only 40.7% and 46.7% reported that they consider it important to habitually wash their hands after changing cat litter and gardening, respectively.

### 3.5. Associations Between Sociodemographic Factors and Preventive Behaviours Towards Toxoplasmosis

Table 5 summarises the associations between sociodemographic factors and preventive behaviours towards toxoplasmosis. Participants educated to university level were more likely to consider it important to habitually wash their hands after gardening since becoming pregnant than those with a lower level of education (53.0% vs. 17.6%; *p* = 0.017). Participants who had experienced 1–3 previous deliveries were also more likely to habitually wash their hands after gardening than those who had experienced ≥8 previous deliveries (52.0% vs. 12.5%; *p* < 0.001). Furthermore, participants with no history of abortion were more likely to habitually wash their hands after gardening than those who had undergone one abortion (54.2% vs. 32.3%; *p* = 0.001). Moreover, participants who had ever seen, heard, or read any information about toxoplasmosis were more likely to habitually wash their hands after changing cat litter than their counterparts who had not (41.3% vs. 31.7%; *p* = 0.025). Similarly, participants who had ever owned cats were more likely to consider it important to habitually wash their hands after changing cat litter than those who had not (90.9% vs. 36.3%; *p* = 0.002). Furthermore, participants who had experienced 1–3 previous deliveries were more likely to habitually wash their hands after handling raw meat than those with ≥8 previous deliveries (93.0% vs. 87.5%; *p* = 0.047). Additionally, participants who had ever been tested for toxoplasmosis were more likely to habitually wash their hands after handling raw meat than those who were not sure whether they had ever been tested for toxoplasmosis (95.0% vs. 75.0%; *p* = 0.046). Finally, students were more likely to eat rare meat since becoming pregnant than housewives (27.8% vs. 2.5%; *p* = 0.008).

### 3.6. Associations Between Knowledge Levels About Toxoplasmosis and Preventive Behaviours

The analysis of the associations between knowledge levels and preventive behaviours, as presented in Table 6, revealed a significant association among the participants for one specific preventive behaviour, namely, habitual handwashing after gardening. Among the participants who reported habitually engaging in this practice, 14.3% displayed a good level of knowledge about toxoplasmosis, compared with those who reported not habitually adhering to this behaviour. This association was statistically significant (*p* < 0.001).

## 4. Discussion

The aim of the present study was to evaluate the knowledge of toxoplasmosis and corresponding preventive behaviours among pregnant Saudi women in Jeddah, Saudi Arabia. The study found that the majority of participants exhibited poor knowledge of toxoplasmosis but practised adequate preventive behaviours.

The observed poor knowledge about toxoplasmosis is consistent with previous studies, which indicated an overall low level of knowledge regarding toxoplasmosis, its risk factors, and its prevention among pregnant women in various countries. In Kuwait, approximately 50% of the participating pregnant women displayed a low level of basic knowledge of toxoplasmosis; nonetheless, over 90% of participants avoided most high-risk activities during pregnancy [23]. In Malaysia, 69.5% of participating pregnant women were found to have a low level of knowledge of toxoplasmosis, but most of them (99.8%) practised preventive behaviours [28]. Moreover, studies conducted in Poland [29], Malaysia, the Philippines, and Thailand [22] reported that most participants had limited knowledge of toxoplasmosis but practised good preventive behaviours. This could be related to a general awareness of other diseases caused by other microorganisms rather than *T. gondii* specifically. Contrary to this finding, a previous study in Saudi Arabia suggested that only 26.8% of pregnant women knew about the forms of *T. gondii* infection transmission; moreover, most of them did not practise the necessary preventive behaviours [24].

In the current study, less than half of the participants (46.7%) reported ever having seen, heard, or read any information about toxoplasmosis. This finding aligns with previous research involving pregnant women spanning diverse geographical contexts. For example, a study conducted in the U.S. state of Minnesota found that less than half of the participants had heard of toxoplasmosis [30], while research performed in Casablanca, Morocco, found that only 41.3% of the participants had prior knowledge of toxoplasmosis through reading or hearing about it [21]. This low level of awareness potentially places these women at higher risk for *T. gondii* infection. Interestingly, despite this lack of awareness, Saudi Arabia currently implements a routine antenatal screening programme in healthcare centres, which includes testing for *Toxoplasma* antibodies during the first trimester of pregnancy [31]. This is further evidenced by our finding that only 14.8% of participants reported awareness of being tested for toxoplasmosis, although all pregnant women in Saudi Arabia undergo mandatory toxoplasmosis screening as part of routine antenatal care. This discrepancy between available screening procedures and low awareness highlights a critical gap in public health communication and emphasises the need for improved patient education about routine antenatal screening procedures. Moreover, this study found that 59.3% of participants recognised the importance of washing fruits and vegetables as a preventive measure. Studies in Saudi Arabia have highlighted the significance of waterborne and foodborne transmission routes for *T. gondii*, where key risk factors identified included the consumption of raw and unwashed fruits and vegetables, and eating at restaurants [11,25]. Globally, transmission routes have evolved over time, with recent outbreaks linked to oocysts in water, soil, and produce [4]. These findings emphasise the need for improved public health education on toxoplasmosis prevention, particularly focusing on proper food handling, water safety, and vegetable washing practices in Saudi Arabia.

The present study also showed that pregnant women who had experienced 4–7 previous deliveries exhibited a significantly higher level of knowledge about toxoplasmosis than other participants. This is consistent with a recent study reporting a significant higher knowledge score among pregnant women with more than one child compared with women with one or no children [32]. Furthermore, another study conducted in Poland to evaluate the knowledge of toxoplasmosis among pregnant women identified a strong relationship between a greater knowledge of toxoplasmosis symptoms and number of children [33]. Moreover, this study indicated that pregnant women aged 31–40 years had a good level of knowledge about toxoplasmosis. The statistical analysis revealed that women in this age group were significantly less likely to have poor knowledge (OR = 0.38; 95% CI: 0.16–0.89, *p* = 0.049) compared to other age groups. This finding aligns with previous studies that revealed a low prevalence of infection among individuals aged 35–44 years [34,35], suggesting that good knowledge in this age group may contribute to lower infection rates.

Nonetheless, most participants appeared to practise adequate preventive behaviours, with 90.4% reporting habitually washing their hands after handling raw meat and 86.7% reporting that they did not eat rare meat. These findings are in line with another study where most of the participating pregnant women (>83%) reported washing their hands after gardening, changing cat litter, and handling raw meat [22]. Contrary to this finding, another work found that most of the participating pregnant women (77%) did not respond correctly to the question of whether *T. gondii* infections could be prevented by avoiding the consumption of undercooked meat [32]. In this context, it has been estimated that 30–60% of *T. gondii* infections occur through the consumption of undercooked meat [36]. Therefore, adopting the behaviour of avoiding the consumption of undercooked meat could reduce the disease burden.

The present data revealed that several characteristics, including having experienced 1–3 previous deliveries, having no history of abortion, having seen, heard, or read information about toxoplasmosis, having been tested for toxoplasmosis, and having owned cats, were significantly associated with the responses of participants towards preventive behaviours, including habitual handwashing after gardening, changing cat litter, and handling raw meat. Thus, these participants avoided the common risk factors known to cause *T. gondii* infection, despite their inadequate levels of knowledge. This finding is supported by several previous studies, which reported that pregnancy itself affects the attitudes and knowledge of women towards *T. gondii* infection [37,38].

This study has several limitations. Firstly, the cross-sectional design inherently prevents the establishment of temporal relationships between exposure and outcome. Secondly, the focus on the city of Jeddah alone may limit the generalisability of the findings to pregnant women in other Saudi Arabian cities, although many of the obtained insights may be broadly applicable. Thirdly, the utilised questionnaire, which was administered as a face-to-face interview, is subject to social desirability bias. Fourthly, the study design could be improved by including questions about water sources and vegetable handling knowledge and preventive behaviours. Fifthly, although all pregnant women in the study underwent mandatory toxoplasmosis screening as part of routine antenatal care, these antibody test results were not incorporated into the analysis. Future research should consider including these serological results to provide a more comprehensive understanding of the relationship between toxoplasmosis status and pregnancy outcomes. Finally, because of the relatively small sample size, the results may be underpowered to detect significant associations. Therefore, future studies on this topic would benefit from larger sample sizes to improve precision and allow for a more nuanced analysis. Despite these limitations, this study still provides valuable insights because it is the first to examine toxoplasmosis knowledge and behaviour among pregnant women in Jeddah. Furthermore, it provides data that can be compared with findings from other regions of Saudi Arabia and elsewhere, contributing to a more comprehensive understanding of regional variations in toxoplasmosis awareness and prevention practices.

## 5. Conclusions

Although most of the pregnant women surveyed in this study demonstrated poor knowledge about *T. gondii* infection, they generally reported practising adequate preventive behaviours. This discrepancy highlights the need for targeted educational interventions. Prenatal education focusing on common risk factors for toxoplasmosis and improved personal hygiene, combined with routine serological testing, could significantly reduce the disease burden and help prevent congenital toxoplasmosis.

The findings underscore the importance of implementing comprehensive training programmes for medical personnel. These programmes should cover additional clinical and preventive measures for congenitally transmitted diseases, including toxoplasmosis, and provide guidance on effectively educating pregnant women about both preventive measures and routine antenatal screening tests. Early pregnancy classes need to include toxoplasmosis education using communication tools such as mobile applications and social media platforms to deliver pregnancy health information. Partnerships with local media can play a crucial role in improving public health information about toxoplasmosis prevention.

Future research should include multicentre studies across diverse regions of Saudi Arabia to provide a more representative sample of pregnant women, while employing larger sample sizes to enhance the statistical power. Moreover, comparative analyses between different regions, socioeconomic groups, and healthcare settings should be performed to reveal important variations in toxoplasmosis awareness and prevention practices. Additionally, future studies should include the knowledge of water and vegetable transmission routes in questionnaire designs to address gaps in Saudi Arabian *T. gondii* epidemiology related to these transmission routes. Furthermore, collaborating with the Saudi Ministry of Health to develop evidence-based guidelines for prenatal health communication and recommend the inclusion of these strategies in national maternal health initiatives is suggested. These efforts align with Saudi Arabia’s 2030 vision to improve quality of life across the population. The implementation of these recommendations by public health authorities could lead to significant advancements in maternal and foetal health across the kingdom.

## Figures and Tables

**Table 1:** Sociodemographic characteristics of the participants (*n* = 135).

Variable	Frequency (*N*)	Percentage (%)
Age (years)		
≤30	77	57.0
31–40	47	34.8
41–50	11	8.1
Educational level		
Illiterate/intermediate education	17	12.6
University	100	74.1
Postgraduate	18	13.3
Profession		
Housewife	80	59.3
Student	18	13.3
Paid employee	37	27.4
Trimester of pregnancy		
First	12	8.9
Second	46	34.1
Third	77	57.0
Number of previous deliveries (*n* = 133)		
1–3	100	75.2
4–7	25	18.8
≥8	8	6.0
History of abortion		
No	83	61.4
Once	31	23.0
More than once	21	15.6
Ever seen, heard, or read any information about toxoplasmosis		
No	60	44.4
Yes	63	46.7
Not sure	12	8.9
Ever been tested for toxoplasmosis		
No	103	76.3
Yes	20	14.8
Not sure	12	8.9
Ever owned cats		
No	124	91.9
Yes	11	8.1

**Table 2:** Knowledge about toxoplasmosis among the participants (*n* = 135).

Question	Correct Answer	
	Response	*N* (%)
General information		
Toxoplasmosis is caused by an infection	Yes	66 (48.9)
Toxoplasmosis is caused by a poison	No	68 (50.4)
Toxoplasmosis (*T. gondii*) is shed in the faeces of infected cats	Yes	82 (60.7)
Toxoplasmosis (*T. gondii*) is sometimes found in raw or undercooked meat	Yes	44 (32.6)
Risk factors		
People may get toxoplasmosis by changing cat litter	Yes	78 (57.8)
People may get toxoplasmosis by eating undercooked meat	Yes	6 (4.4)
People may get toxoplasmosis by gardening without gloves	Yes	38 (28.1)
Symptoms and timing of infection		
Pregnant women may develop serious complications after infection with *T. gondii*	Yes	93 (68.9)
Unborn and/or newborn children may develop serious complications after infection with *T. gondii*	Yes	57 (42.2)
Toxoplasmosis in a pregnant woman may cause fever and feeling like you have a ‘flu’	Yes	73 (54.1)
Toxoplasmosis in a pregnant woman may cause swollen glands (lymph nodes)	Yes	24 (17.8)
Toxoplasmosis in a pregnant woman may cause no symptoms	Yes	56 (41.5)
Toxoplasmosis (*T. gondii*) can only be passed from a pregnant woman to her foetus if she becomes newly infected during that pregnancy	Yes	74 (54.8)
Toxoplasmosis (*T. gondii*) is rarely passed from a pregnant woman to her foetus if she was infected before becoming pregnant	Yes	42 (31.1)
A baby with toxoplasmosis may have no signs of illness at birth but may develop symptoms later	Yes	44 (32.6)
A baby with toxoplasmosis may have vision problems	Yes	30 (22.2)
A baby with toxoplasmosis may be treated with medicine	Yes	91 (67.4)
Preventive knowledge		
Ways to avoid toxoplasmosis include feeding cat dry or commercial cat food and not letting it kill and eat rodents	Yes	67 (49.6)
Ways to avoid toxoplasmosis include avoiding stray cats	Yes	111 (82.2)
Ways to avoid toxoplasmosis include letting someone else change a cat’s litter box	Yes	94 (69.6)
Ways to avoid toxoplasmosis include making sure a cat’s litter box is changed daily	Yes	100 (74.1)
Toxoplasmosis can be prevented by cooking meat well until no pink is seen and the juices run clear	Yes	81 (60.0)
Toxoplasmosis can be prevented by thoroughly washing and/or peeling all fruits and vegetables before eating them	Yes	80 (59.3)
Toxoplasmosis can be prevented by cleaning all cutting boards and utensils thoroughly after each use	Yes	81 (60.0)

**Table 3:** Associations between sociodemographic factors and knowledge about toxoplasmosis among the participants (*n* = 135).

Variable	Knowledge Level		Crude Odds Ratio (95% CI)	Adjusted Odds Ratio (95% CI)	*p* Value *
	Poor/Fair *N* = 105 *N* (%)	Good *N* = 30 *N* (%)			
Age (years)					
≤30 (*n* = 77)	64 (83.1)	13 (16.9)	1.0	1.0	
31–40 (*n* = 47)	31 (66.0)	16 (34.0)	0.39 (0.17–0.92)	0.38 (0.16–0.89)	
41–50 (*n* = 11)	10 (90.9)	1 (9.1)	2.03 (0.24–17.27)	1.83 (0.21–15.69)	0.049
Educational level				Removed from final model	
Illiterate/intermediate education (*n* = 17)	13 (76.5)	4 (23.5)	1.0		
University (*n* = 100)	79 (79.0)	21 (21.0)	1.16 (0.34–3.92)		
Postgraduate (*n* = 18)	13 (72.2)	5 (27.8)	0.80 (0.17–3.67)		0.965
Profession				Removed from final model	
Housewife (*n* = 80)	61 (76.3)	19 (23.7)	1.0		
Student (*n* = 18)	13 (72.2)	5 (27.8)	0.81 (0.26–2.56)		
Paid employee (*n* = 37)	31 (83.8)	6 (16.2)	1.61 (0.58–4.44)		0.501
Trimester of pregnancy				Removed from final model	
First (*n* = 12)	11 (91.7)	1 (8.3)	1.0		
Second (*n* = 46)	35 (76.1)	11 (23.9)	0.29 (0.03–2.50)		
Third (*n* = 77)	59 (76.6)	18 (23.4)	0.30 (0.04–2.47)		0.310
Number of previous deliveries (*n* = 133)	*N* = 103	*N* = 30		Removed from final model	
1–3 (*n* = 100)	80 (80.0)	20 (20.0)	1.0		
4–7 (*n* = 25)	16 (64.0)	9 (36.0)	0.44 (0.17–1.15)		
≥8 (*n* = 8)	7 (87.5)	1 (12.5)	1.75 (0.20–15.05)		0.044
History of abortion				Removed from final model	
No (*n* = 83)	64 (77.1)	19 (22.9)	1.0		
Once (*n* = 31)	22 (71.0)	9 (29.0)	0.73 (0.29–1.84)		
More than once (*n* = 21)	19 (90.5)	2 (9.5)	2.82 (0.60–13.21)		0.494
Ever seen, heard, or read any information about toxoplasmosis				Removed from final model	
No (*n* = 60)	43 (71.7)	17 (28.3)	1.0		
Yes (*n* = 63)	53 (84.1)	10 (15.9)	2.10 (0.87–5.04)		
Not sure (*n* = 12)	9 (75.0)	3 (25.0)	1.19 (0.29–4.92)		0.487
Ever been tested for toxoplasmosis				Removed from final model	
No (*n* = 103)	78 (75.7)	25 (24.3)	1.0		
Yes (*n* = 20)	17 (85.0)	3 (15.0)	1.82 (0.49–6.71)		
Not sure (*n* = 12)	10 (83.3)	2 (16.7)	1.60 (0.33–7.81)		0.636
Ever owned cats				Removed from final model	
No (*n* = 124) ^a^	98 (79.0)	26 (21.0)	1.0		
Yes (*n* = 11)	7 (63.6)	4 (36.4)	0.46 (0.13–1.71)		0.372

* Chi-square test; ^a^ reference category; CI = confidence interval.

**Table 4:** Self-reported preventive behaviours towards toxoplasmosis among the participants (*n* = 135).

Questions	Yes	No	Not Sure
Since becoming pregnant, do you consider it important to habitually wash your hands after gardening?	63 (46.7)	15 (11.1)	57 (42.2)
Since becoming pregnant, do you consider it important to habitually wash your hands after changing cat litter?	55 (40.7)	6 (4.4)	74 (54.8)
Since becoming pregnant, do you consider it important to habitually wash your hands after handling raw meat?	122 (90.4)	8 (5.9)	5 (3.7)
Since becoming pregnant, do you eat rare meat?	13 (9.6)	117 (86.7)	5 (3.7)

**Table 5:** Associations between sociodemographic factors and preventive behaviours towards toxoplasmosis among the participants (*n* = 135).

Variable	Habitually Washing Hands After Gardening			*p* Value	Habitually Washing Hands After Changing Cat Litter			*p* Value	Habitually Washing Hands After Handling Raw Meat			*p* Value	Eating Rare Meat			*p* Value
	Yes *N* = 63 *N* (%)	No *N* = 15 *N* (%)	Not Sure *N* = 57 *N* (%)		Yes *N* = 55 *N* (%)	No *N* = 6 *N* (%)	Not Sure *N* = 74 *N* (%)		Yes *N* = 122 *N* (%)	No *N* = 8 *N* (%)	Not Sure *N* = 5 *N* (%)		Yes *N* = 13 *N* (%)	No *N* = 117 *N* (%)	Not Sure *N* = 5 *N* (%)	
Age (years)																
≤30 (*n* = 77)	39 (50.6)	7 (9.1)	31 (40.3)		31 (40.3)	5 (6.5)	41 (53.2)		67 (87.0)	6 (7.8)	4 (5.2)		9 (11.7)	64 (83.1)	4 (5.2)	
31–40 (*n* = 47)	20 (42.6)	4 (8.5)	23 (48.9)	0.069	16 (34.0)	1 (2.1)	30 (63.8)		44 (93.6)	2 (4.3)	1 (2.1)		4 (8.5)	43 (91.0)	0 (0.0)	
41–50 (*n* = 11)	4 (36.4)	4 (36.4)	3 (27.3)		8 (72.7)	0 (0.0)	3 (27.3)	0.120	11 (100)	0 (0.0)	0 (0.0)	0.596	0 (0.0)	10 (90.9)	1 (9.1)	0.310
Educational level																
Illiterate/intermediate education (*n* = 17)	3 (17.6)	4 (23.5)	10 (58.8)		7 (41.2)	0 (0.0)	10 (58.8)		17 (100)	0 (0.0)	0 (0.0)		0 (0.0)	17 (100)	0 (0.0)	
University (*n* = 100)	53 (53.0)	11 (11.0)	36 (36.0)	0.017	44 (100)	6 (100)	50 (50.0)		88 (88.0)	7 (7.0)	5 (5.0)		12 (12.0)	83 (83.0)	5 (5.0)	
Postgraduate (*n* = 18)	7 (38.9)	0 (0.0)	11 (61.1)		4 (22.2)	0 (0.0)	14 (77.8)	0.193	17 (94.4)	1 (5.6)	0 (0.0)	0.521	1 (5.6)	17 (94.4)	0 (0.0)	0.297
Profession																
Housewife (*n* = 80)	36 (45.0)	10 (12.5)	34 (42.5)		33 (41.3)	2 (2.5)	45 (56.3)		76 (95.0)	2 (2.5)	2 (2.5)		2 (2.5)	75 (93.8)	3 (3.8)	
Student (*n* = 18)	9 (50.0)	2 (11.1)	7 (38.9)		73 (38.9)	2 (11.1)	9 (50.0)		16 (88.9)	1 (5.6)	1 (5.6)		5 (27.8)	12 (66.7)	1 (5.6)	
Paid employee (*n* = 37)	18 (48.6)	3 (8.1)	16 (43.2)	0.961	15 (40.5)	2 (5.4)	20 (54.1)	0.611	30 (81.1)	5 (13.5)	2 (5.4)	0.165	6 (16.2)	30 (81.1)	1 (2.7)	0.008
Trimester of pregnancy																
First (*n* = 12)	9 (75.0)	0 (0.0)	3 (25.0)		6 (50.0)	0 (0.0)	6 (50.0)		11 (91.7)	0 (0.0)	1 (8.3)		2 (16.7)	10 (83.3)	0 (0.0)	
Second (*n* = 46)	20 (43.5)	4 (8.7)	22 (47.8)		19 (41.3)	2 (4.3)	25 (54.3)		42 (91.3)	2 (4.3)	2 (4.3)		1 (2.2)	43 (93.5)	2 (4.3)	
Third (*n* = 77)	34 (44.2)	11 (14.3)	32 (41.6)	0.220	30 (39.0)	4 (5.2)	43 (55.8)	0.907	69 (89.6)	6 (7.8)	2 (2.6)	0.668	10 (13.0)	64 (83.1)	3 (3.9)	0.282
Number of previous deliveries (*n* = 133)																
1–3 (*n* = 100)	52 (52.0)	7 (7.0)	41 (41.0)		42 (42.0)	5 (5.0)	53 (53.0)		93 (93.0)	3 (3.0)	4 (4.0)		8 (8.0)	88 (88.0)	4 (4.0)	
4–7 (*n* = 25)	9 (36.0)	1 (4.0)	15 (60.0)		6 (24.0)	1 (4.0)	18 (72.0)		21 (84.0)	4 (16.0)	0 (0.0)		4 (16.0)	21 (84.0)	0 (0.0)	
≥8 (*n* = 8)	1 (12.5)	6 (75.0)	1 (12.5)	<0.001	5 (62.5)	0 (0.0)	3 (37.5)	0.292	7 (87.5)	0 (0.0)	1 (12.5)	0.047	0 (0.0)	8 (100)	0 (0.0)	0.451
History of abortion																
No (*n* = 83)	45 (54.2)	6 (7.2)	32 (38.6)		36 (43.4)	4 (4.8)	43 (51.8)		74 (89.2)	6 (7.2)	3 (3.6)		8 (9.6)	73 (88.0)	2 (2.4)	
Once (*n* = 31)	10 (32.3)	2 (6.5)	19 (61.3)		10 (32.3)	1 (3.2)	20 (64.5)		28 (90.3)	1 (3.2)	2 (6.5)		3 (9.7)	26 (83.9)	2 (6.5)	
More than once (*n* = 21)	8 (38.1)	7 (33.3)	6 (28.6)	0.001	9 (42.9)	1 (4.8)	11 (52.4)	0.820	20 (95.2)	1 (4.8)	0 (0.0)	0.709	2 (9.5)	18 (85.7)	1 (4.8)	0.892
Ever seen, heard, or read any information about toxoplasmosis																
No (*n* = 60)	31 (51.7)	3 (5.0)	26 (43.3)		9 (31.7)	3 (5.0)	38 (63.3)		53 (88.3)	4 (6.7)	3 (5.0)		5 (8.3)	51 (85.0)	4 (6.7)	
Yes (*n* = 63)	24 (38.1)	10 (15.9)	29 (46.0)		26 (41.3)	3 (4.8)	34 (54.0)		58 (92.1)	3 (4.8)	2 (3.2)		6 (9.5)	56 (88.9)	1 (1.6)	
Not sure (*n* = 12)	8 (66.7)	2 (16.7)	2 (16.7)	0.089	10 (83.3)	0 (0.0)	2 (16.7)	0.025	11 (91.7)	1 (8.3)	0 (0.0)	0.889	2 (16.7)	10 (83.3)	0 (0.0)	0.490
Ever been tested for toxoplasmosis																
No (*n* = 103)	2 (50.5)	8 (7.8)	43 (41.7)		40 (38.8)	5 (4.9)	58 (56.3)		94 (91.3)	5 (4.9)	4 (3.9)		9 (8.7)	90 (87.4)	4 (3.9)	
Yes (*n* = 20)	8 (40.0)	5 (25.0)	7 (35.0)		11 (55.0)	1 (5.0)	8 (40.0)		19 (95.0)	0 (0.0)	1 (5.0)		1 (5.0)	19 (95.0)	0 (0.0)	
Not sure (*n* = 12)	3 (25.0)	2 (16.7)	7 (58.3)	0.109	4 (33.3)	0 (0.0)	8 (66.7)	0.560	9 (75.0)	3 (25.0)	0 (0.0)	0.046	3 (25.0)	8 (66.7)	1 (8.3)	0.228
Ever owned cats																
No (*n* = 124)	57 (46.0)	15 (12.1)	52 (41.9)		45 (36.3)	6 (4.8)	73 (58.9)		112(90.3)	8 (6.5)	4 (3.2)		10 (8.1)	109(87.9)	5 (4.0)	
Yes (*n* = 11)	6 (54.5)	0 (0.0)	5 (45.5)	0.468	10 (90.9)	0 (0.0)	1 (9.1)	0.002	10 (90.9)	0 (0.0)	1 (9.1)	0.439	3 (27.3)	8 (72.7)	0 (0.0)	0.101

**Table 6:** Associations between knowledge levels about toxoplasmosis and preventive behaviours towards toxoplasmosis among the participants (*n* = 135).

Preventive Behaviour	Knowledge Level			*p* Value *
	Poor *N* = 61 *N* (%)	Fair *N* = 44 *N* (%)	Good *N* = 30 *N* (%)	
Since becoming pregnant, you habitually wash your hands after gardening				
No (*n* = 15)	13 (87.7)	2 (13.3)	0 (0.0)	
Yes (*n* = 63)	32 (50.8)	22 (34.9)	9 (14.3)	
Not sure (*n* = 57)	16 (28.1)	20 (35.1)	21 (36.8)	<0.001
Since becoming pregnant, you habitually wash your hands after changing cat litter				
No (*n* = 6)	5 (83.3)	1 (16.7)	0 (0.0)	
Yes (*n* = 55)	26 (47.3)	19 (34.5)	10 (18.2)	
Not sure (*n* = 74)	30 (40.5)	24 (32.4)	20 (27.0)	0.251
Since becoming pregnant, you habitually wash your hands after handling raw meat				
No (*n* = 8)	5 (62.5)	1 (12.5)	2 (25.0)	
Yes (*n* = 122)	52 (42.6)	43 (35.2)	27 (22.1)	
Not sure (*n* = 5)	4 (80.0)	0 (0.0)	1 (20.0)	0.290
Since becoming pregnant, you eat rare meat				
No (*n* = 117)	53 (45.3)	38 (32.5)	26 (22.2)	
Yes (*n* = 13)	3 (23.1)	6 (46.2)	4 (30.8)	
Not sure (*n* = 5)	5 (100)	0 (0.0)	0 (0.0)	0.071

* Chi-square test.

## Data Availability

Data are contained within the article.
